# Modelling the effects of climate and human factor on Lassa fever distribution in Ondo State Nigeria

**DOI:** 10.1007/s00484-025-02996-3

**Published:** 2025-08-11

**Authors:** Temitope Emmanuel Arotolu, Josephine Olayinka-Olagunju, Adekunle A. Dosumu

**Affiliations:** 1 College of Wildlife Resources and Protected Areas, Northeast Forestry University, Harbin, 150040 Heilongjiang Province P. R. China; 2 Department of Animal and Environmental Biology, Adekunle Ajasin University Akungba Akoko, Akungba Akoko, Ondo State Nigeria; 3 Department of Biosciences, School of Science and Technology, Nottingham Trent University, Clifton Lane, Nottingham, NG11 8NS UK

**Keywords:** Lassa fever, Mastomys, MaxEnt, PCA, Ondo state, AUC, Bioclimatic variables

## Abstract

Lassa fever is an acute viral haemorrhagic disease caused by the Lassa virus. Transmission to humans primarily occurs through direct contact with Mastomys rats or via the ingestion of food or usage of household items contaminated with the urine or faeces of infected rats. The MaxEnt algorithm was used to estimate the distribution of Lassa fever based on data on the incidence of the disease, ecogeographic features, and human factors. Principal component analysis (PCA) was used to mitigate multicollinearity among the environmental variables. The model’s accuracy was evaluated using the area under the receiver operating characteristic curve (AUC-ROC). The prevalence of Lassa fever is anticipated to be substantially affected by human factors (population density, roads, built-settlement, poverty), climatic variables (Prec11, Tmean01, Bio7, Bio12), and altitude. The model distribution map revealed that Owo, Ose, Akure North, Akure South, Akoko South-West, Akoko South-East, Akoko North-East, Ifedore, Idanre, Ondo, and Akoko North-West are very suitable regions. Our suitability map identifies hotspots, aiding public health officials in resource distribution to mitigate the current Lassa fever epidemic in Ondo State, Nigeria.

**Supplementary Information:**

The online version contains supplementary material available at 10.1007/s00484-025-02996-3.

## Introduction

Lassa fever is an acute viral haemorrhagic disease caused by the Lassa virus, which belongs to the arenavirus family (Richmond and Baglole 2003). Humans typically contract the Lassa virus by coming into contact with food or household objects tainted with the urine or faeces of infected *Mastomys natalensis*, the principal reservoir (van Meer 2024). The primary risk factors in Nigeria consist of exposure to surfaces or food contaminated with rodent urine or faeces, frequently occurring during the open-air post-harvest drying of grains or the handling of diseased rats for eating (Dalhat et al. 2022). Infection may arise upon exposure to the bodily fluids of an infected person. Individuals infected with Lassa fever are not transmissible until the onset of their symptoms (Ilori et al. 2019).

Lassa fever is endemic in West Africa, particularly in Nigeria, where annual outbreaks happen from December to March. Individuals infected with the Lassa fever virus commonly exhibit acute symptoms including headaches, mild fever, sore throat, myalgia, vomiting, and diarrhoea. In serious cases, symptoms may escalate to respiratory distress, shock, emesis, and haemorrhaging from the mouth, nasal passages, vagina, or gastrointestinal tract (WHO 2017; Ezeomah et al. 2019). Lassa fever poses a concern to humans due to its fatal consequences and complications. Lassa fever may result in hearing impairment, with around one-third of cases exhibiting varying degrees of deafness. Deafness may manifest in both moderate and severe instances of Lassa fever. In numerous instances, the auditory impairment is irreversible (Reuben et al. 2020). Additionally, there is a significant chance of miscarriages in infected pregnant women, with around 95% of foetuses failing to survive (CDC 2025). Signs and symptoms of Lassa fever generally manifest 1 to 3 weeks post-infection, and this extended incubation period allows infected individuals in endemic areas to travel both domestically and internationally, potentially leading to an epidemic (Inegbenebor et al. 2010).

In Nigeria, where the case-fatality ratio attains 20%, it constitutes over half of the diagnoses (Ipadeola et al. 2020). The Lassa fever belt in southern Nigeria, encompassing Ose Local Government Area in Ondo State adjacent to Edo State and extending westward to areas like Owo, is deemed the most endemic region, responsible for approximately 71% of the nation’s total cases (Gibb et al. 2017; Siddle et al. 2018; Aloke et al. 2023). The seasonal maxima of this viral haemorrhagic fever occur throughout the dry season from November to April, predominantly in West Africa (WHO 2016). The Lassa fever virus is presently classified among the viruses with the greatest potential for animal-to-human transmission and is also one of the most probable to emerge beyond its endemic region (Fichet-Calvet and Rogers 2009).

The interconnectedness of West African nations and the facilitation of human mobility heighten the potential of transnational disease transmission. Disease surveillance systems and adequate outbreak response are essential public health measures in cross-border migration (Kakaī et al. 2020). Research by Tuite et al. evaluated case exportation during the 2018 Lassa fever outbreak, indicating that countries near Nigeria should remain vigilant for potential Lassa virus importation and guarantee timely epidemic response (Tuite et al. 2019). In contrast, countries beyond Africa, such as the United Kingdom, have recorded incidents of Lassa fever (Wise 2025). Imported cases from Sierra Leone were documented in the Netherlands (Overbosch et al. 2020).

As of March 11, 2025, Nigeria has reported Lassa fever outbreak with 535 confirmed cases and 98 fatalities across 14 states since the beginning of the year, resulting in an 18.3% case fatality rate (NCDC 2025). The disease has become prevalent in the states of Ondo, Bauchi, Edo, Taraba, and Ebonyi (Grace et al. 2021; Nyenke et al. 2022; Asogun et al. 2025). The consistent rise of Lassa fever cases in Ondo State highlights the state’s significance in the broader epidemiology of the disease in Nigeria, raising substantial public health concerns (Ejikeme et al. 2021; Cadmus et al. 2023). Efforts have intensified to combat the persistent outbreak of Lassa fever in Ondo State through prior research on the influence of seasonal variations and climatic factors, such as temperature and precipitation, on the frequency of Lassa fever, as well as the evaluation of various ecological indicators’ impacts on its prevalence in Ondo State (Cadmus et al. 2023; Cadmus et al. 2025). This study was conducted to assess the ecological suitability and predictive factors of Lassa fever transmission in Ondo State.

## Materials and methods

### Study location/area

**Fig. 1:**
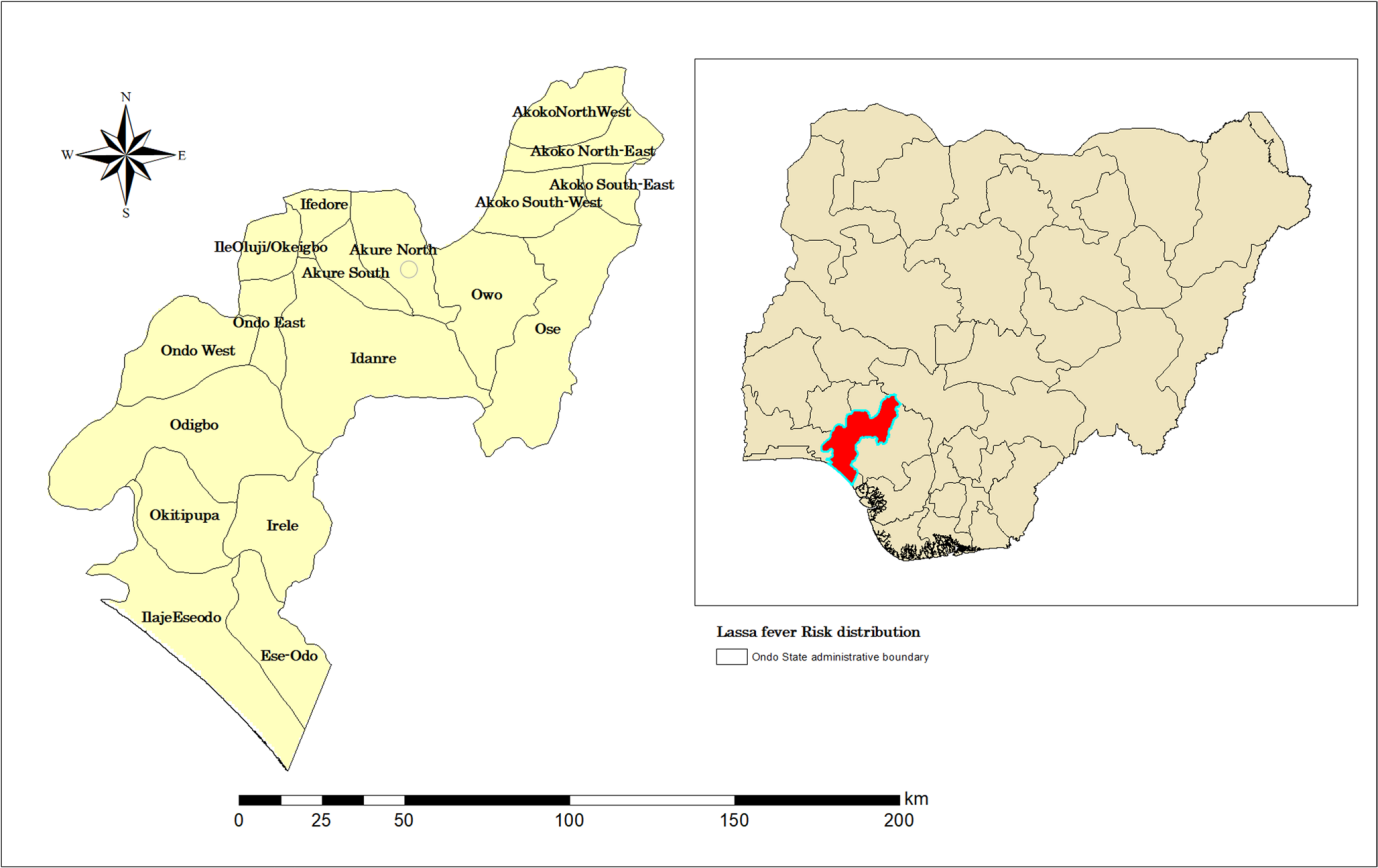
Study area indicating (**A**) Ondo state (**B**) Nigeria (study area in red)

The research area is Ondo State, located in Southwest Nigeria. The state is situated between longitudes 4°30’ and 6° East of the Greenwich Meridian, and latitudes 5°45’ and 8°15’ North of the Equator, placing it wholly within the tropical zone. It is bordered to the North by Ekiti/Kogi State, to the East by Edo State, to the West by Oyo and Ogun States, and to the South by the Atlantic Ocean. Ondo State encompasses an area of around 14,788.723 square kilometres (5,709 square miles), categorizing it as one of Nigeria’s fairly large states (Fig. 1). The elevation of Ondo State varies considerably owing to its varied topography, covering coastal zones, forested areas, and hilly landscapes. The mean elevation is roughly 250 m above sea level (m.a.s.l). Certain regions of the state, notably the Idanre Hills, ascend to elevations above 1,000 m above sea level. The natural landscape of Ondo State includes coastal wetlands, tropical rainforests, and highland regions, rendering it ecologically and economically significant. It is distinguished by its agricultural output, especially in cocoa, lumber, and palm oil production.

### Occurrence data and Environmental variables collection

This study used a total of one hundred and forty-three (*n* = 143) cases of Lassa fever, derived from geographic information obtained from published literature and the Nigeria Centre for Diseases Control (NCDC) Weekly Lassa Fever Situation Report spanning 2017 to 2025. Sixty-eight meteorological parameters and elevation were obtained from WorldClim version 2.1 for the period 1970 to 2000 at a resolution of 30 arc-seconds. The human population density (2000–2020), built settlement/housing, and distance to road are publicly accessible at gratis via the WorldPop Dataverse Repository and the WorldPop project website (https://hub.worldpop.org/). Data on population density were obtained from the World Bank’s database (https://maps.worldbank.org/en/data/datatopics/poverty-portal/home).

### Data preprocessing

**Fig. 2:**
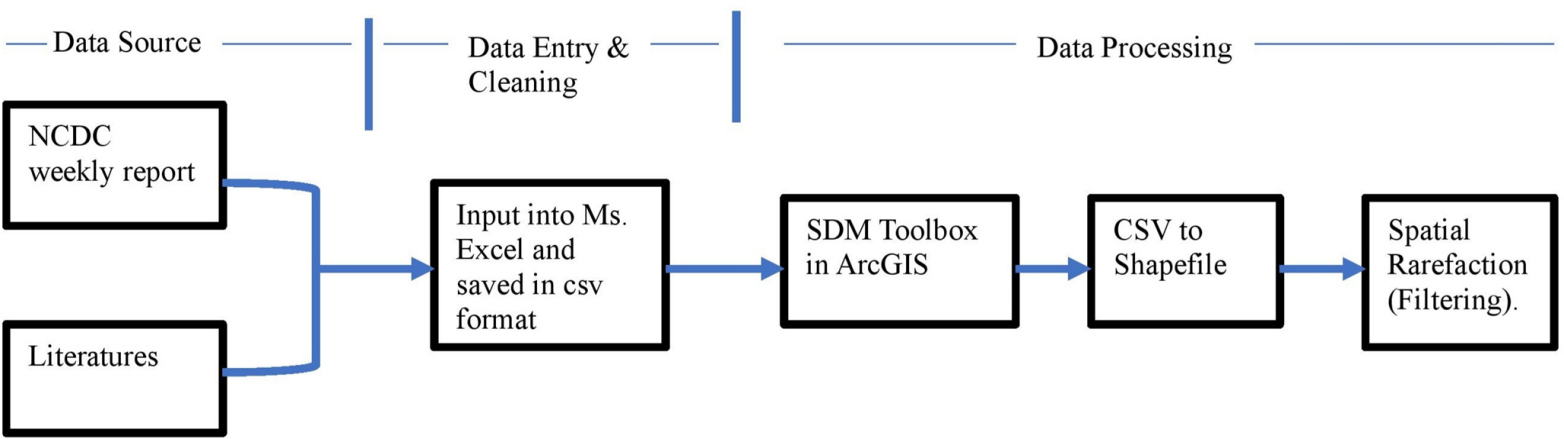
Flowchart for the Filtering process to reduce spatial autocorrelation

**Fig. 3:**
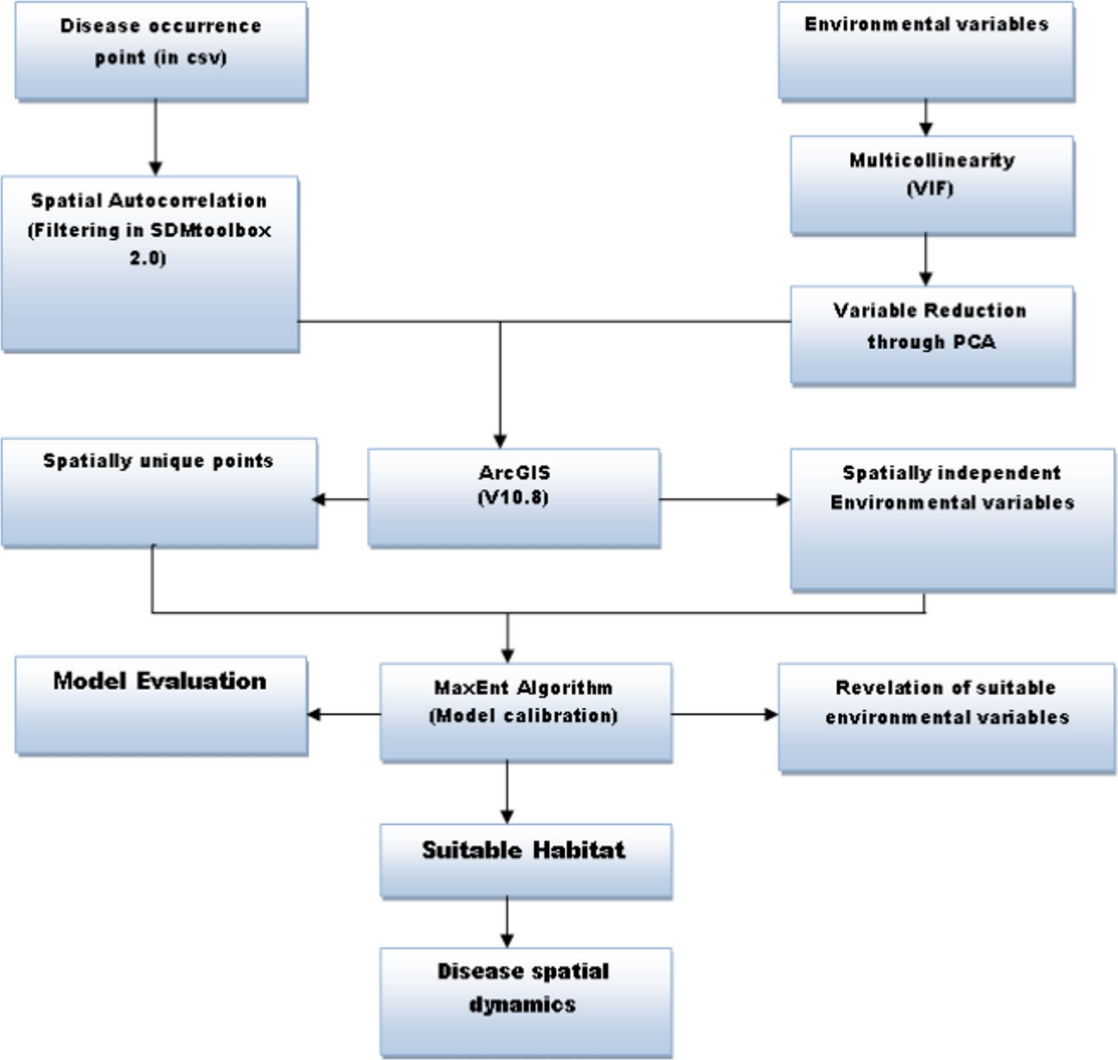
Flowchart for the modelling process with the potential variables

Data cleaning involved the deletion of records containing geocoordinate inaccuracies, which were corrected and verified using Microsoft Excel. Spatial autocorrelation was mitigated by filtering the Lassa fever occurrence data with SDM toolbox v1.1c coupled with ArcGIS v10.6. Data filtering was executed by establishing a minimum distance of 10 km between each pair of disease occurrence locations (Fig. 2). In the initial filtering, we applied the default distance setting (natural break), which ensures that the minimum distance between two adjacent points as 5 km, 10 km, 15 km, 20 km and 30 km (Fekede et al. 2019; Zeng et al. 2021; Arotolu et al. 2022a). Our model AUC performed poorly when the minimum distance was set at 5 km apart, but improved greatly when 10 km was set apart, and there was no significant difference between distance of 10–30 km model AUC. Therefore, we chose 10 km as the minimum distance between each pair of occurrence points. This mitigates geographical sampling biases. The occurrence points were subsequently saved as comma-separated values (CSV) and imported into ArcGIS v10.6 for editing. The data were converted to UTM-WGS-1984 using standard parameters and resampled to a resolution of 30 arc-seconds (van Gils et al. 2014). Sixty-eight meteorological parameters and elevation were obtained from WorldClim version 2.1 for the period 1970 to 2000 at a resolution of 30 arc-seconds. The human population density (2000–2020), built settlement/housing, and distance to road are publicly accessible at gratis via the WorldPop Dataverse Repository and the WorldPop project website (https://hub.worldpop.org/). Data on population density were obtained from the World Bank’s database (https://maps.worldbank.org/en/data/datatopics/poverty-portal/home). All spatial data were pre-processed and computed in ArcGIS 10.6, projected in UTM-WGS-1984 using conventional settings or resampled to 30 arc seconds (van Gils et al. 2014) (Fig. 3).

### Model fitting and development

The occurrence points were filtered by defining the shortest possible distance between each pair of locations using the SDM Toolbox v1.1c in ArcGIS 10.6 to reduce spatial autocorrelation (Arotolu et al. 2022a). The reduction of variables and potential multicollinearity among the environmental factors were assessed by principal component analysis (PCA) utilizing the Statistical Package for Social Sciences software (SPSS v22.0). Multicollinearity can breach statistical assumptions and modify model predictions (Heikkinen et al. 2006). The uncorrelated variables were utilized for MaxEnt modelling. All environmental rasters were clipped to the research region utilizing the “extract by mask” tool, ensuring that the “processing extent” is consistent across all variables within the environmental settings to confirm that all files encompass the same area (Jácome et al. 2019). Multicollinearity was diminished for both climate and non-climate predictors. Initially, we incorporated the climate predictor variables based on the PCA. We utilized the variables when eigenvalues above 1.0, adhering to the scree plot criterion or broken stick ‘stopping rule’ for PCA in item-level factoring (Zeng et al. 2021). To save climatic predictor variables for future use in the MaxEnt model, superfluous loading was suppressed, and the factor patterns of climatic variables were rotated. Secondly, the filtered occurrences of Lassa fever and environmental factors were used as input data in the MaxEnt model (van Gils et al. 2014). The area under the receiver operating characteristic (ROC) curve (AUC) is used to ensure the robustness of the MaxEnt model (Austin 2007). AUC-ROC values characterize the model at several levels of precision: (1) Excellent model: 0.9–1; (2) Good model: 0.8–0.9; (3) Fair model: 0.7–0.8; (4) Poor model: < 0.7. The models calculate the area under the curve (AUC) values and compare the variable percentages of two regions. We systematically removed the predictors with minimal contribution and elevated standard deviation (SD) until we identified the final environmental factors (Gao et al. 2021). We utilized the definitive environmental factors for model prediction. The collinearity assessment is conducted for all the variables ultimately acquired. The variance inflation factor (VIF) is computed for all final variables, with a value below 10 signifying low and acceptable multicollinearities using SPSS (Duque-Lazo et al. 2016). The MaxEnt model version 3.4.1 was executed using filtered Lassa fever occurrence data and 10,000 random background points, which represent the environmental conditions’ distribution within the study area. We selected a random seed to ensure the model selects different sets of presence records for training and testing in each replication. We used the MaxEnt model with auto features, a convergence threshold of 0.00001, a maximum number of background points of 10,000, and a regularisation multiplier of 1. The dataset comprised training data (75%) and test data (25%). Two subsets of the data were used to construct and validate the models through 100 bootstrap replicates (Phillips and Dudík 2008).

### Model evaluation

The criterion used to evaluate model performance is an important part of the model validation methods. To measure the relative contribution of each environmental variable to the predictive model, a Jackknife manipulation was performed. The area under the ROC curve (AUC) is a threshold-independent criterion that is calculated by graphing true positives vs. false positives for a variety of predictive probability thresholds. Currently, the AUC is regarded as the best measure for determining model success using presence/absence data. Our model was assessed using the area under the ROC curve (AUC-ROC) (Li et al. 2020). A Jackknife test was used to assess how each environmental variable contributed to the predictive model. The Jackknife test identifies the most effective single variable for predicting the distribution. Additionally, a smooth response curve served as a quality criterion (Fekede et al. 2019; Arotolu et al. 2022b). The three methods; AUC-ROC, Jackknife test, and the variable response curves are optimal for assessing model performance. AUC values range from 0 to 1, where 1 signifies perfect discrimination and 0.5 corresponds to random performance. Additionally, we utilize correctly classified instances (CCI) (Fielding and Bell 1997) as the threshold-dependent criterion for the model. The thresholds for this criterion are established by the maximum of the sensitivity-specificity summation (Gao et al. 2023).

## Results

The spatial rarefaction of Lassa fever incidence data obtained 84 out of 143 occurrence records, ensuring a minimum distance of 10 km between each record. PCA reduced the 68 climate variables to 28 independent variables. The independent variables were incorporated into the MaxEnt algorithms. Using the MaxEnt techniques, we excluded variables with minimal contribution rates using backward stepwise elimination method and ultimately identified Twelve meteorological factors. Following the integration of the twelve bioclimatic variables and additional environmental variables into the model. The predictors with minimal contribution and those exhibiting large standard deviation, as determined by visual analysis of the response curves, were excluded. Ultimately, we acquired built settlement (Housing), human population density, proximity to roads, poverty prevalence, November precipitation (Prec11), annual temperature range (BIO5-BIO6) (Bio7), elevation, mean temperature in January (Tmean01), and annual precipitation (Bio12) for the final model.

**Fig. 4:**
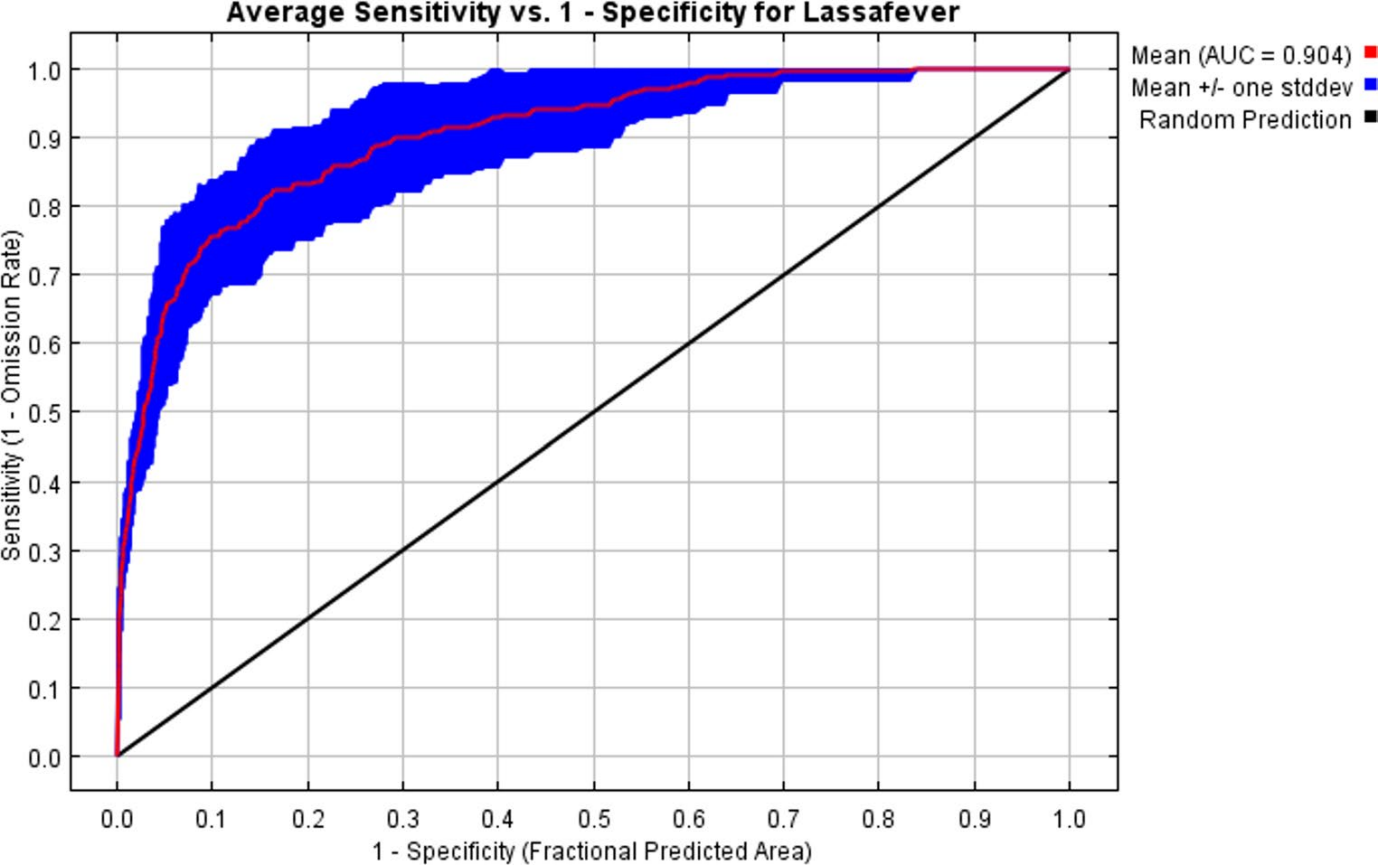
Average receiver operating characteristics and related area under the curve (AUC) of the 100 bootstrap replicates

**Fig. 5:**
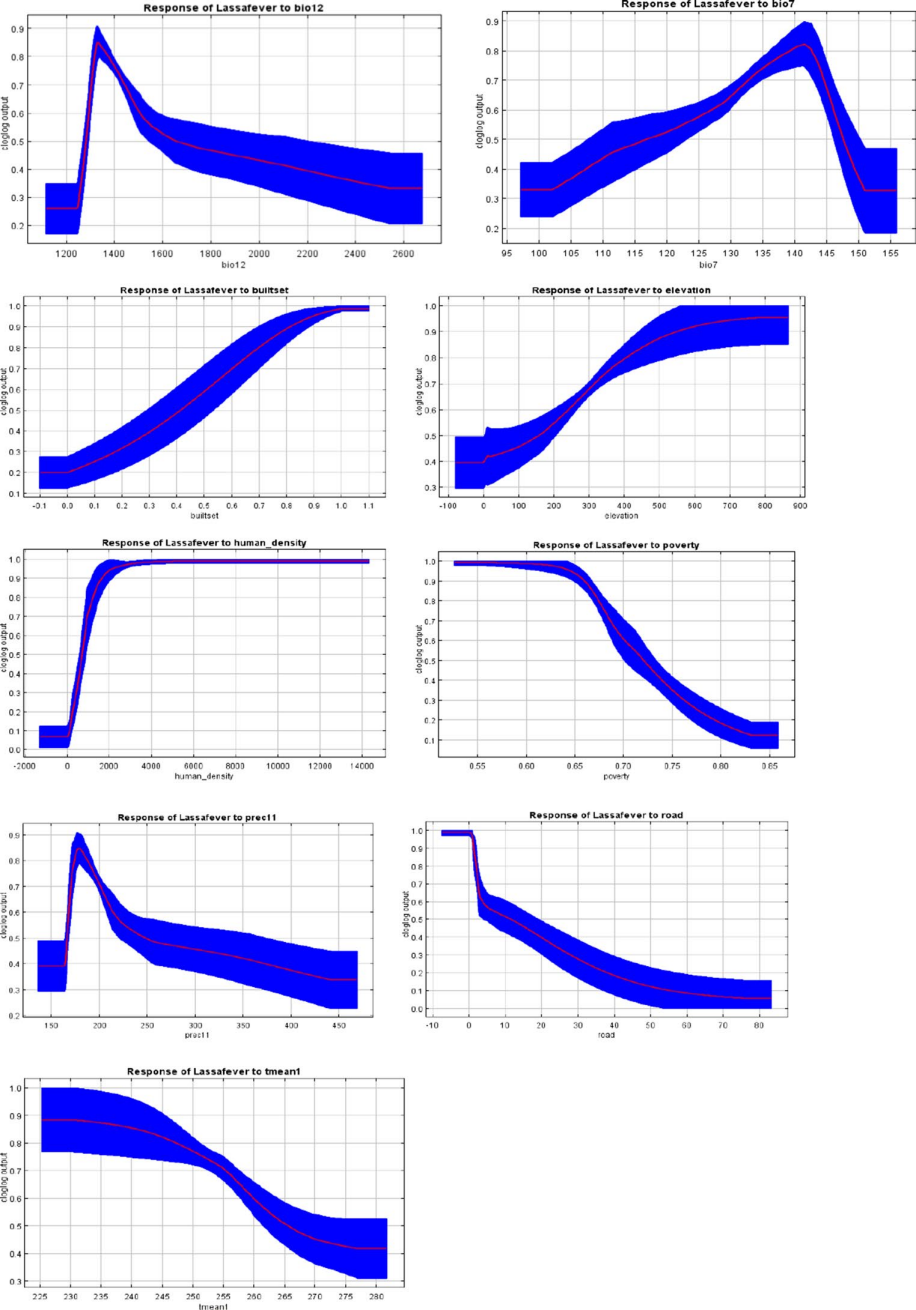
Response curves of continuous predictor variables. The red lines indicate the mean values while the blue lines denote the standard deviation

**Fig. 6:**
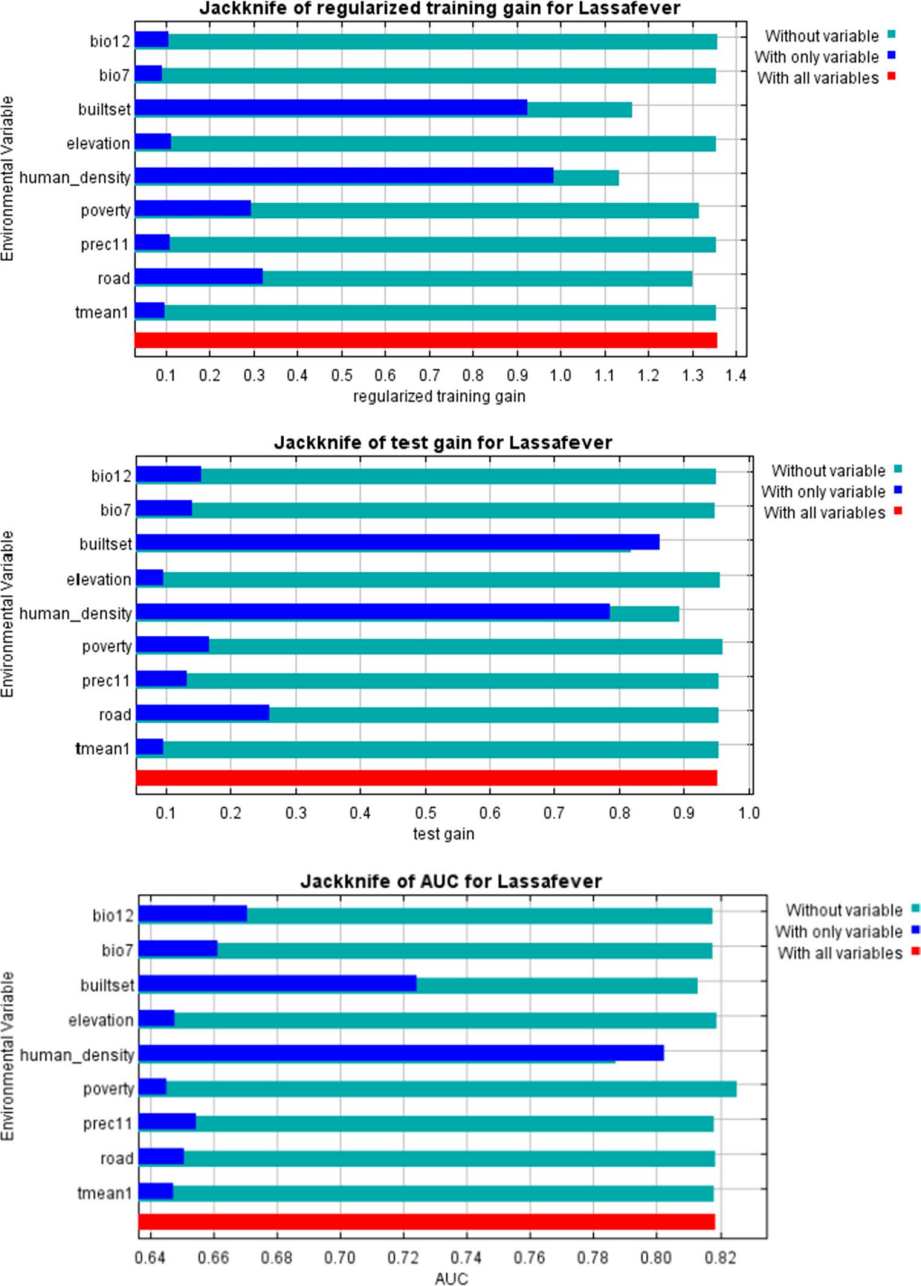
Summary of the Jackknife analysis performed to determine importance of each environmental variable

**Fig. 7:**
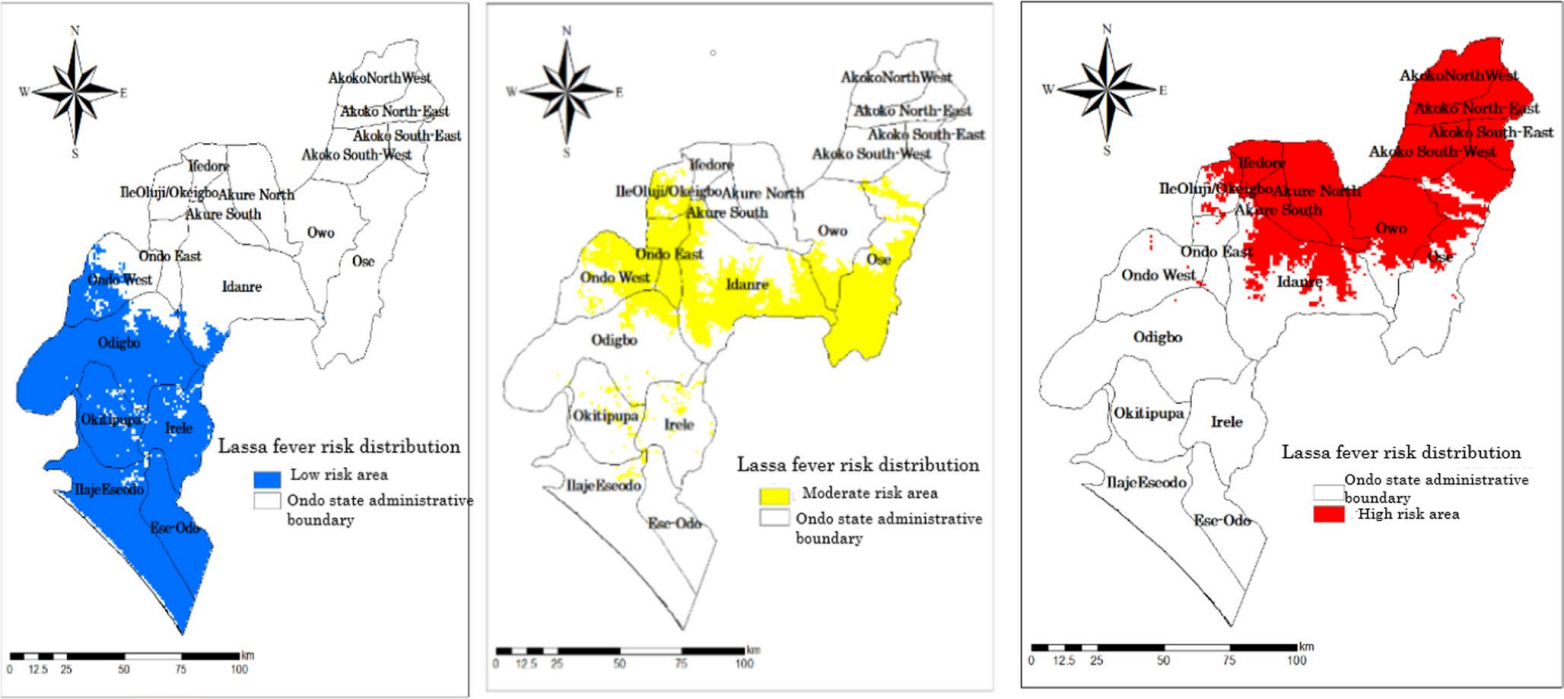
**Fig. 7 ** The environmental suitability map of Lassa fever in Ondo State. The blue colour indicate low suitable area, yellow colour indicate moderate suitable area, and red indicate high suitable area

Figure 4 illustrates the response curves of the various predictors, while Table 1 presents the relative contributions of each predictor. The prediction model was validated using the computed AUC for evaluation. The calculated AUC and correctly classified instances (CCI) are 0.904 and 0.900, respectively, signifying optimal predictive capability and affirming the robustness of our model (Fig. 4 and 5). Jackknife analysis conducted to ascertain the significance of each environmental variable (Fig. 6). The jackknife test reveals that the omission of any of these nine variables affects the model’s regularization gain, test gain, and AUC value (Table 1). The human population density and built settlement exhibited the most substantial training gains when analysed as individual environmental variables. Conversely, the four bioclimatic variables exhibited the lowest values. Moreover, the minimal training gain occurred when the human population density was omitted from the model, whereas the model exhibited the maximum gain when elevation and the four bioclimatic variables were eliminated (Fig. 6). Figure 7 illustrates the high-risk zones identified by the MaxEnt model.

**Table 1:** Jackknife contribution table of the environmental predictors

Variable	Percent contribution (%)	Permutation importance
Built settlement/Housing	49.1	10.6
Human population density	39.9	63.2
Distance to road	4.2	8.6
Poverty prevalence	3.1	2.3
Prec11	1.2	2.8
Bio7	1.1	4.1
Elevation	0.9	4.3
Tmean1	0.4	2.8
Bio12	0.3	1.4

*Prec11- November precipitation. *Bio7- Temperature Annual Range (BIO5-BIO6)

*Tmean1- Mean Temperature in January. *Bio12- Annual precipitation

The built-settlement and human population density exhibited the most significant test gains when used as the sole environmental variable, while Elevation and Mean Temperature in January (Tmean01) demonstrated the least test gain. Our model had the lowest AUC when the variable of human population density was omitted. In contrast, the model achieved the maximum AUC when solely using human population density as the variable. The MaxEnt algorithm model was used to identify high-risk locations for Lassa fever in the Ondo State (Fig. 7). The MaxEnt prediction result correlates with the relative occurrence rate, indicating the habitat compatibility of each pixel. Owo, Ose, Akure North, Akure South, Akoko South-West, Akoko South-East, Akoko North-East, Ifedore, Idanre, Ondo, and Akoko North-West exhibit the highest likelihood of Lassa disease incidence in Ondo State. The Ondo West, Odigbo, and Irele local government areas have a moderate risk likelihood for Lassa fever. The likelihood of occurrence is negligible in the southern region of Ondo State.

## Discussion

This study investigates the spatial analysis of climatic and anthropogenic factors influencing the spread of Lassa fever in Ondo State, Nigeria, the state with the highest burden of the disease. Numerous studies on Lassa fever, encompassing epidemiological analysis and trends (Salu et al. 2020; Dalhat et al. 2022; Cadmus et al. 2023) as well as distribution mapping (Redding et al. 2021; Al-Mustapha et al. 2024; Odetokun et al. 2024), have been undertaken in Nigeria. We examined nine years of recorded Lassa fever outbreaks using MaxEnt algorithms to assess the influence of environment and human factors on the distribution of Lassa disease in Ondo State, a primary epicentre in Nigeria. The environmental predictors in our model may indicate the predictors directly, have mixed influence (Madueme and Chirove 2024), or serve as proxies for them. This study corroborates prior research indicating that the distribution of Lassa fever is primarily influenced by built settlements (Redding et al. 2021; Cadmus et al. 2023), human population density (Abdullahi et al. 2020; Dalhat et al. 2022), proximity to roads (Cadmus et al. 2023), poverty prevalence (Osho et al. 2020), and climatic factors (Fichet-Calvet and Rogers 2009; Clegg 2013; Redding et al. 2021).

Built settlements, housing characteristics, and domestic practices also affect rat population density, establishing conducive environmental circumstances in homes with appropriate rodent habitats that can promote virus transmission (Bonwitt et al. 2017; Mariën et al. 2018). Elevated population density and inadequate sanitation can foster conditions conducive to rodent proliferation, hence invariably heightening the risk of Lassa fever outbreaks (Mariën et al. 2018; Gobir et al. 2020; Usuwa et al. 2020; Gomerep et al. 2022). Increased population densities in metropolitan regions may enhance human-to-human transmission and influence the location of rodent reservoirs. Inadequate sanitation and waste management, resulting from substandard urban development and elevated population density, may create environments conducive to rodent proliferation, hence heightening the risk of human contact (Ter Meulen et al. 1996; Abdullahi et al. 2020; Dalhat et al. 2022). Distance to road as predicted in our model is thought to be connected with Lassa fever incidence because it influences changes in land use patterns, human activities, and rodent reservoir distribution (Bonwitt et al. 2017).

The high annual precipitation of approximately 1200–1400 mm in our model aligns with the findings of Fichet-Calvet and Rogers (2009), which indicate that the distribution of human Lassa fever outbreaks and cases correlates with annual precipitation ranging from 1200 to 1500 mm (Fichet-Calvet and Rogers 2009). Lassa Virus (Arenaviruses) are enveloped viruses that exhibit limited resilience to high temperatures and low humidity conditions. Consequently, certain climatic conditions are anticipated to affect the survival of viruses in the environment, either adversely or favourably. This effect may explain the correlation between Lassa fever distribution in West Africa and precipitation, as previously mentioned (Fichet-Calvet and Rogers 2009). Temperature and Lassa fever incidence have an intricate relationship that can be impacted by multiple variables including as rainfall, humidity, and land use patterns. Our study agrees with study by Patz and Olson 2006 that Lassa fever virus responds to temperature and humidity variations, and that outbreaks have been connected to these factors. For example, droughts or extreme weather events might cause rats to move into metropolitan areas, thereby increasing the risk of human exposure (Patz and Olson 2006). In Ondo State, Lassa fever incidence shows seasonal changes, with peaks during the dry season and fewer cases during the rainy season, indicating that climatological conditions and agricultural labour patterns may influence rodent-human contact (Leach et al. 2017; Cadmus et al. 2023). Previous study by (Nchom et al. 2021) suggests that warmer temperatures can increase rat populations’ reproductive rates, increasing the number of sick animals and potentially enhancing the danger of transmission of Lassa fever to humans. Understanding these temperature-related patterns might help create strategies for minimizing Lassa fever and lowering the danger of outbreaks in Ondo State.

Our model identified elevation as a significant predictive factor for the incidence of Lassa fever in Ondo State. Our MaxEnt model indicated that moderate altitude (above 500 m.a.s.l) is a critical factor for the occurrence of Lassa fever, corroborating the findings of Cadmus et al. (Cadmus et al. 2023). Our research indicates that bioclimatic conditions, human population density, and associated activities are distinct elements influencing the spatial distribution of Lassa disease in Ondo State, Nigeria.

Environmental factors such as land use and land cover, are thought to influence the ecology of rats, which act as the principal reservoirs for the Lassa virus. Conventional, areas frequently feature open areas designated as waste disposal sites, structures that facilitate rodent entry, and underdeveloped infrastructure. Housing type and poverty prevalence could foster inadequate household waste management, lack of good storage materials, inability to afford healthcare, and tightly-knit communities in these areas may lead to the clustering of Lassa fever. Other notable ecological factors, including population density, land use patterns, and sanitary conditions, influence Lassa fever incidences. High population density and Indiscriminate waste disposal can encourage conditions conducive to rodent growth, hence increasing the likelihood of Lassa fever epidemics (Abdullahi et al. 2020; Dalhat et al. 2022). Housing factors and household practices influence rat density, which may impact Lassa fever transfer to humans (Bonwitt et al. 2017). Evidence from previous study by Zhang et al. 2020 and McLay et al. 2019 opined that nighttime light exposure may influence rodent populations and can alter mouse behaviour. Nighttime light draws rats to illuminated zones for feeding and nesting, and potentially increase their vulnerability to Lassa virus infection (McLay et al. 2019; Zhang et al. 2020).

Our study revealed high-risk regions for Lassa fever in the northern parts of Ondo State, specifically Owo, Ose, Akure North, Akure South, Akoko South-West, Akoko South-East, Akoko North-East, Ifedore, Idanre, Ondo, and Akoko North-West. The geographical proximity of the anticipated high-risk regions to Edo State, a prominent epicentre of Lassa fever, may suggest the potential for transboundary spread of the disease. The Ondo West, Odigbo, and Irele local government areas have a moderate likelihood of Lassa fever risks. The likelihood of occurrence is negligible in the southern region of Ondo State. Adesina et al. (2023) shown in their study on Lassa virus cross-species transmission in the endemic Edo-Ondo state axis that there is an East-West migration of the virus across southwestern Nigeria (Adesina et al. 2023).

The current study has identified high-risk areas and factors in the northern part of the state; nevertheless, it also has a few limitations. In addition to the lack of distribution of rodent population density in our model. We recommend a comprehensive investigation of Lassa fever in humans and animals to examine their correlation. Additionally, socio-cultural determinants, including traditional food storage methods, availability of healthcare services, and communal living conditions, are believed to influence the aggregation of Lassa fever cases (Mariën et al. 2018; Gobir et al. 2020; Usuwa et al. 2020; Gomerep et al. 2022) and should be incorporated into future research to elucidate the factors driving Lassa fever in Ondo State and Nigeria as a whole.

## Conclusion

This study explored the spatial distribution of Lassa disease in Ondo State, the state with the greatest burden of Lassa fever in Nigeria. Our model demonstrated that both climatic and anthropogenic factors promote the survival of the Lassa fever virus in Ondo State. The risk distribution map identified the northern and elevated regions of Ondo State as the most ecologically suited areas. Our suitability map identifies hotspots, aiding public health officials in resource distribution to alleviate the current Lassa fever epidemic in Ondo State, Nigeria.

### Recommendation

We therefore recommend effective public hygiene to deter rodents from infiltrating homes. Also, effective strategies encompass the storage of grain and other foodstuffs in rodent-proof containers, the disposal of refuse at a distance from homes, the maintenance of cleanliness within households, and the confinement of cats indoors. Currently, there is no vaccination accessible for human use. The antiviral medication ribavirin may serve as an effective treatment for Lassa fever if administered early in the illness.

## Supplementary Information

Below is the link to the electronic supplementary material.

Supplementary Material 1 (DOC 91.0 KB)

## Data Availability

All data are available and the sources are listed in the article.

## Abbreviations

LF: Lassa fever
WHO: World Health Organisation
NCDC: Nigerian Centre for Disease Control
MaxEnt: Maximum entropy
UTM-WGS-1984: Universal Transverse Mercator (UTM) coordinate system and the World Geodetic System 1984 (WGS84) datum
GIS: Geographic information system
AUC: Area under the curve
ROC: Receiver operating characteristics
CCI: Correctly classified instances
SPSS: Statistical package for social sciences
PCA: Principal component analysis
m.a.s.l: Meters above sea level
SD: Standard deviation
