# Supplementary material for: Modelling the effects of climate and human factor on Lassa fever distribution in Ondo State Nigeria
Source: Int J Biometeorol. 2025 Aug 11;69(10):2553–65. doi: 10.1007/s00484-025-02996-3 (PMC12540567; doi:10.1007/s00484-025-02996-3)
Supplement: Supplementary file 1 — Supplementary Material 1 (DOC 91.0 KB) [file 484_2025_2996_MOESM1_ESM.doc]

Appendix 1: Lassa fever occurrence data

| Species | Longitude | Latitude |
| --- | --- | --- |
| Lassafever | 5.7813 | 6.9381 |
| Lassafever | 5.77368 | 7.4228 |
| Lassafever | 5.8997 | 7.297 |
| Lassafever | 5.6754 | 7.4458 |
| Lassafever | 4.89356 | 6.89592 |
| Lassafever | 5.89816 | 7.4305 |
| Lassafever | 5.7669 | 7.5248 |
| Lassafever | 4.713 | 6.763 |
| Lassafever | 4.45286 | 6.72121 |
| Lassafever | 5.0807 | 6.9582 |
| Lassafever | 5.5696 | 7.1911 |
| Lassafever | 5.1328 | 7.054 |
| Lassafever | 5.013 | 7.4946 |
| Lassafever | 5.1067 | 7.3497 |
| Lassafever | 4.81036 | 6.24956 |
| Lassafever | 3.319 | 6.683 |
| Lassafever | 5.1901 | 7.250771 |
| Lassafever | 4.841694 | 7.10002 |
| Lassafever | 5.586673 | 6.782935 |
| Lassafever | 4.9137 | 6.4262 |
| Lassafever | 5.0108 | 7.40243 |
| Lassafever | 4.80406 | 6.35266 |
| Lassafever | 4.8676 | 7.2017 |
| Lassafever | 5.083 | 7.167 |
| Lassafever | 4.7226 | 7.171 |
| Lassafever | 4.95497 | 7.2944 |
| Lassafever | 4.8676 | 6.7947 |
| Lassafever | 4.7795 | 6.5025 |
| Lassafever | 4.9559 | 7.0881 |
| Lassafever | 4.96808 | 6.98879 |
| Lassafever | 5.3704 | 7.2579 |
| Lassafever | 5.6681 | 7.1322 |
| Lassafever | 5.6879 | 7.0155 |
| Lassafever | 5.75943 | 7.64314 |
| Lassafever | 5.25919 | 7.39452 |
| Lassafever | 5.3871 | 1.198 |
| Lassafever | 5.0005 | 6.25754 |

Appendix 2: Ranking the number of predictor variables in the principal component analysis.

| **PC Ranking** | **EigenValues** | |
| --- | --- | --- |
|  | **Percent of EigenValues** | **Accumulative of EigenValues** |
| 1 | 78.2678 | 78.2678 |
| 2 | 10.4128 | 88.6806 |
| 3 | 5.1842 | 93.8648 |
| 4 | 2.1066 | 95.9714 |
| 5 | 2.0195 | 97.9909 |
| 6 | 1.0055 | 98.9964 |

Appendix 3: Bioclimatic, elevation, classical meteorological and human impact variables used for initial modeling in maxent software (T- Temperature and P – Precipitation).

| **Label** | **Variable description** | **Units** |  |
| --- | --- | --- | --- |
| **1.     Bioclimatic variables** | | |  |
| Bio1 | Annual Mean T | °C |  |
| Bio2 | Mean Monthly Diurnal Range (Tmax - Tmin) | °C |  |
| Bio3 | Isothermally (BIO2/BIO7) x 100 | Index |  |
| Bio4 | T Seasonality (Standard Deviation) | °C |  |
| Bio5 | Max T of Warmest Month | °C |  |
| Bio6 | Min T of Coldest Month | °C |  |
| Bio7 | T Annual Range (BIO5-BIO6) | °C |  |
| Bio8 | Mean T of Wettest Quarter | °C |  |
| Bio9 | Mean T of Driest Quarter | °C |  |
| Bio10 | Mean T of Warmest Quarter | °C |  |
| Bio11 | Mean T of Coldest Quarter | °C |  |
| Bio12 | Annual P | mm |  |
| Bio13 | P of Wettest Month | mm |  |
| Bio14 | P of Driest Month | mm |  |
| Bio15 | P Seasonality (Coefficient of Variation) | Fraction |  |
| Bio16 | P of Wettest Quarter | mm |  |
| Bio17 | P of Driest Quarter | mm |  |
| Bio18 | P of Warmest Quarter | mm |  |
| Bio19 | P of Coldest Quarter | mm |  |
| **2.     Elevation** | | |  |
| Alt | Elevation | m a.s.l. |  |
| **3.     Classical meteorological variables** | | |  |
| Monthly P | Monthly P (n=12) | mm |  |
| Monthly  Tmean, Tmin, Tmax | Monthly mean, minimum and maximum T (n=36) | °C |  |
|  | **4. Human Impact** |  |  |
| Population | Human population density | Individual/km2 | |
| Road | Distance to Road | Km |  |
| Housing | Built Settlement | - |  |
| Poverty | Poverty prevalence | - |  |

Appendix 4: Environmental variables used for the final model

| **Variables** | **Type** | **Unit** | **Source** |
| --- | --- | --- | --- |
| Built settlement (Housing) | Continuous | - | WorldPop^a^ |
| Human population density | Continuous | persons/km^2^ | WorldPop^a^ |
| Distance to road | Continuous | km | WorldPop^a^ |
| Poverty prevalence | Continuous | - | Worldbank^b^ |
| November precipitation | Continuous | mm | WorldClim^c^ |
| Temperature annual range | Continuous | °C | WorldClim^c^ |
| Elevation | Continuous | m.a.s.l | WorldClim^c^ |
| Mean Temperature in January | Continuous | °C | WorldClim^c^ |
| Annual precipitation | Continuous | mm | WorldClim^c^ |

^a^sources: WorldPop Dataverse Repository (<http://worldpop.org.uk/data/>).

^b^Source: World Bank Database (https://maps.worldbank.org/en/data/datatopics/poverty-portal/home).

^c^Source: Worldclim 1.4 (https://[worldclim.org](http://www.worldclim.org/)) at 30 arcsecond resolution.
